# Supplementary material for: Modeling glioblastoma heterogeneity as a dynamic network of cell states
Source: Mol Syst Biol. 2021 Sep 16;17(9):e10105. doi: 10.15252/msb.202010105 (PMC8444284; doi:10.15252/msb.202010105)
Supplement: Supplementary file 6 — Source Data for Figure 5 [file MSB-17-e10105-s004.zip › Figure5A_sourcedata/GSEA_3071/nosigdb_stateVI.GseaPreranked.1622795169056/RICHARDS_VERHAAK_CANCERCELL_2010_PRONEURAL.html]

Details for gene set RICHARDS\_VERHAAK\_CANCERCELL\_2010\_PRONEURAL[GSEA]

|  || Dataset | state63071 |
| Phenotype | NoPhenotypeAvailable |
| Upregulated in class | na\_neg |
| GeneSet | RICHARDS\_VERHAAK\_CANCERCELL\_2010\_PRONEURAL |
| Enrichment Score (ES) | -0.6196158 |
| Normalized Enrichment Score (NES) | -2.8309283 |
| Nominal p-value | 0.0 |
| FDR q-value | 0.0 |
| FWER p-Value | 0.0 |
Table: GSEA Results Summary

  

Fig 1: Enrichment plot: RICHARDS\_VERHAAK\_CANCERCELL\_2010\_PRONEURAL      
 Profile of the Running ES Score & Positions of GeneSet Members on the Rank Ordered List

  

| PROBE | GENE SYMBOL | GENE\_TITLE | RANK IN GENE LIST | RANK METRIC SCORE | RUNNING ES | CORE ENRICHMENT || 1 | ALCAM |  |  | 7 | 1.185 | 0.0195 | No |
| 2 | FERMT1 |  |  | 76 | 0.794 | 0.0149 | No |
| 3 | ICK |  |  | 848 | 0.369 | -0.1941 | No |
| 4 | ZNF804A |  |  | 946 | 0.353 | -0.2149 | No |
| 5 | RALGPS2 |  |  | 1619 | 0.267 | -0.3981 | No |
| 6 | SRGAP3 |  |  | 1836 | -0.254 | -0.4539 | No |
| 7 | PAK3 |  |  | 1870 | -0.260 | -0.4584 | No |
| 8 | MAPT |  |  | 1922 | -0.267 | -0.4678 | No |
| 9 | PHLPP1 |  |  | 1937 | -0.269 | -0.4669 | No |
| 10 | OLIG2 |  |  | 1968 | -0.274 | -0.4703 | No |
| 11 | TMEFF1 |  |  | 2158 | -0.306 | -0.5176 | No |
| 12 | CLASP2 |  |  | 2286 | -0.330 | -0.5472 | No |
| 13 | BCL7A |  |  | 2359 | -0.346 | -0.5610 | No |
| 14 | ABAT |  |  | 2438 | -0.365 | -0.5762 | No |
| 15 | LRP6 |  |  | 2505 | -0.379 | -0.5878 | No |
| 16 | ATAD5 |  |  | 2574 | -0.392 | -0.5998 | No |
| 17 | STMN1 |  |  | 2646 | -0.411 | -0.6122 | Yes |
| 18 | DCAF7 |  |  | 2663 | -0.414 | -0.6091 | Yes |
| 19 | ARHGEF9 |  |  | 2681 | -0.419 | -0.6063 | Yes |
| 20 | FBXO21 |  |  | 2706 | -0.427 | -0.6053 | Yes |
| 21 | ZNF711 |  |  | 2736 | -0.435 | -0.6055 | Yes |
| 22 | TOPBP1 |  |  | 2774 | -0.448 | -0.6077 | Yes |
| 23 | YPEL1 |  |  | 2781 | -0.449 | -0.6013 | Yes |
| 24 | RAD21 |  |  | 2797 | -0.457 | -0.5972 | Yes |
| 25 | TSPAN3 |  |  | 2828 | -0.465 | -0.5971 | Yes |
| 26 | MLLT11 |  |  | 2850 | -0.476 | -0.5944 | Yes |
| 27 | SATB1 |  |  | 2899 | -0.494 | -0.5988 | Yes |
| 28 | FHOD3 |  |  | 2921 | -0.502 | -0.5956 | Yes |
| 29 | MARCKSL1 |  |  | 2928 | -0.506 | -0.5881 | Yes |
| 30 | CRMP1 |  |  | 3039 | -0.558 | -0.6088 | Yes |
| 31 | TOP2B |  |  | 3059 | -0.567 | -0.6038 | Yes |
| 32 | SOX4 |  |  | 3066 | -0.571 | -0.5951 | Yes |
| 33 | ERBB3 |  |  | 3116 | -0.596 | -0.5980 | Yes |
| 34 | GNG4 |  |  | 3166 | -0.635 | -0.6002 | Yes |
| 35 | DNM3 |  |  | 3185 | -0.648 | -0.5935 | Yes |
| 36 | PFN2 |  |  | 3215 | -0.685 | -0.5892 | Yes |
| 37 | RUFY3 |  |  | 3262 | -0.722 | -0.5890 | Yes |
| 38 | PODXL2 |  |  | 3271 | -0.730 | -0.5780 | Yes |
| 39 | MPPED2 |  |  | 3283 | -0.741 | -0.5676 | Yes |
| 40 | SOX2 |  |  | 3314 | -0.778 | -0.5619 | Yes |
| 41 | GRIA2 |  |  | 3351 | -0.815 | -0.5572 | Yes |
| 42 | TMSB15A |  |  | 3358 | -0.824 | -0.5440 | Yes |
| 43 | MCM10 |  |  | 3360 | -0.825 | -0.5293 | Yes |
| 44 | ZEB2 |  |  | 3395 | -0.881 | -0.5228 | Yes |
| 45 | CDKN1B |  |  | 3426 | -0.951 | -0.5140 | Yes |
| 46 | SOX11 |  |  | 3443 | -0.989 | -0.5005 | Yes |
| 47 | CHD7 |  |  | 3454 | -1.017 | -0.4849 | Yes |
| 48 | DLL3 |  |  | 3460 | -1.030 | -0.4676 | Yes |
| 49 | NRXN2 |  |  | 3462 | -1.034 | -0.4491 | Yes |
| 50 | TTYH1 |  |  | 3481 | -1.067 | -0.4348 | Yes |
| 51 | MMP16 |  |  | 3511 | -1.150 | -0.4221 | Yes |
| 52 | NCAM1 |  |  | 3514 | -1.168 | -0.4014 | Yes |
| 53 | GPM6A |  |  | 3515 | -1.169 | -0.3802 | Yes |
| 54 | MYO10 |  |  | 3516 | -1.170 | -0.3590 | Yes |
| 55 | C1QL1 |  |  | 3520 | -1.186 | -0.3383 | Yes |
| 56 | TMCC1 |  |  | 3538 | -1.259 | -0.3203 | Yes |
| 57 | SLCO5A1 |  |  | 3546 | -1.322 | -0.2982 | Yes |
| 58 | CKB |  |  | 3579 | -1.575 | -0.2786 | Yes |
| 59 | MAP2 |  |  | 3597 | -1.763 | -0.2514 | Yes |
| 60 | ASCL1 |  |  | 3604 | -1.822 | -0.2201 | Yes |
| 61 | BEX1 |  |  | 3608 | -1.883 | -0.1868 | Yes |
| 62 | DGKI |  |  | 3625 | -2.580 | -0.1445 | Yes |
| 63 | C1orf61 |  |  | 3626 | -2.616 | -0.0970 | Yes |
| 64 | FXYD6 |  |  | 3628 | -2.707 | -0.0482 | Yes |
| 65 | DCX |  |  | 3630 | -2.797 | 0.0022 | Yes |
Table: GSEA details [plain text format]

  

Fig 2: RICHARDS\_VERHAAK\_CANCERCELL\_2010\_PRONEURAL: Random ES distribution      
 Gene set null distribution of ES for **RICHARDS\_VERHAAK\_CANCERCELL\_2010\_PRONEURAL**
